# Skin-Lightening Products: Consumer Preferences and Costs

**DOI:** 10.7759/cureus.17245

**Published:** 2021-08-17

**Authors:** Anh-Dao Cheng, Henriette De La Garza, Mayra B.C. Maymone, Vanessa M Johansen, Neelam A Vashi

**Affiliations:** 1 Dermatology, Boston University School of Medicine, Boston, USA; 2 Dermatology, Brown University, Providence, USA

**Keywords:** skin lightening products, skin lightening, skin bleaching, lightening cream, hyperpigmentation

## Abstract

Disorders of skin hyperpigmentation have an appreciable impact on quality of life, carrying social and cultural importance that influences widespread consumer demand for cosmetic lightening products. We sought to investigate product factors that may influence consumer preference when choosing an over-the-counter cream for skin-lightening purposes. The keyword “lightening cream” was searched in the "Beauty and Personal Care category" of online retailer Amazon.com. The top fifth percentile of products was determined by filtering the search results for a minimum of four out of five stars from customer reviews, and then further filtered by products with over 100 reviews. Number of reviews, rating, price per unit, vehicle, application instructions, ingredients, and review attributes were evaluated. Over 2,900 products were catalogued as “lightening cream” on Amazon.com. There were 40 products rated >4 stars and >100 reviews, constituting the top 1.34% of entries. Tocopheryl was present in 58% of the top 40 products, ascorbic acid in 40%, and niacinamide in 20%. Notably, 22.5% of the top 40 products contained no known skin-lightening ingredient. There was a moderate positive correlation (R^2^=0.378) between price and consumer rating, with kojic acid being the most expensive per ounce ($24.89) and salicylic acid being the most highly rated (4.54 stars; $15.00/oz). This study provides insight on the factors influencing the choice, preferences, and satisfaction of consumer that sought Amazon for the purchase of lightening products.

## Introduction

Skin hyperpigmentation disorders have an appreciable impact on quality of life, carrying social and cultural importance that influences widespread consumer demand for cosmetic lightening products [1]. Skin lightening is the process by which substances are used to reduce melanin concentration in dark areas of the skin to even out tone or, in some cases, to achieve an overall lighter complexion. Skin bleaching is an ancient process that can be traced back to 200 Before Common Era (BCE) [2]. From this historical time, skin lightening continues to be a common practice. Today, this can be achieved with soaps, over-the-counter (OTC) and prescription creams, pills, injections, and an assortment of cosmetic procedures. According to the World Health Organization, dangerous skin bleaching without professional counseling has become a public health crisis [3]. Half of the population in Korea, Malaysia, and the Philippines use some kind of skin-lightening treatment, 77% of Nigerian women use skin-lightening products regularly, and 61% of the skin care market in India consists of skin-lightening products [3,4]. Due to economic, comfort, and time constraints, many patients prefer to treat themselves at home instead of seeking professional counsel with a dermatologist. We sought to investigate product factors that may influence consumer preference when choosing an OTC cream for skin-lightening purposes. 

## Materials and methods

In May of 2019, the keyword lightening cream was searched in the "Beauty and Personal Care category" of online retailer Amazon.com, yielding 2,974 results and 40,141 reviews. The top fifth percentile of products was determined by filtering the search results for a minimum of four out of five stars from customer reviews, yielding 148 products, and then further filtered by products with over 100 reviews. The following data were gathered: number of reviews, rating, price per unit, vehicle, application instructions, ingredients, and positive and negative attributes in the reviews. The first five comments listed in Amazon.com’s predetermined top positive and top critical review categories were included in the analysis. For product entries with multiple size options, price per unit was determined by the product size with the most reviews. The following product entries were not included: deodorants, oil, hair serum, hair-lightening products, and toothpaste. Reviewer characteristics, such as skin type, race, age, and/or prior dermatological conditions, were not consistently available on the Amazon.com platform.

## Results

Over 2,900 products were catalogued as “lightening cream” on Amazon.com. There were 40 products rated >4 stars and >100 reviews, constituting the top 1.34% of entries. The average review rating was 4.2 stars out of 5.0, and the average price was $11.73/oz. We reviewed a total of 40,141 reviews with a range of 103 to 6,610 reviews per product (Table 1).

**Table 1:** Summary of factors influencing consumer choices and preferences. **No data. MD, medical doctor.

		Number of mentions, N (%)	Distribution, N (%)	Weighted average review	Average price per ounce
Performance		40 (100)			
	Exclusively positive		8 (20)	4.14	$10.45
	Exclusively negative		0 (0)	**	**
	Mixed		32 (80)	4.26	$12.11
Application		31 (77.5)			
	Exclusively positive		13 (41.9)	4.21	$11.01
	Exclusively negative		10 (32.3)	4.29	$11.27
	Mixed		8 (25.8)	4.26	$14.22
Odor		29 (72.5)			
	Exclusively positive		15 (51.7)	4.24	$15.22
	Exclusively negative		8 (27.6)	4.17	$8.65
	Mixed		6 (20.7)	4.33	$13.06
Ingredients		19 (47.5)			
	Exclusively positive	“Organic” and “natural”	7 (36.8)	4.29	$10.89
	Exclusively negative	“Fake” or “dislike ingredients”	7 (36.8)	4.24	$14.32
	Mixed		5 (26.3)	4.2	$13.26
Side effects					
	Yes	29 (72.5)		4.27	$10.52
	No	11 (27.5)		4.15	$15.11
MD recommended		5 (12.5)		4.11	$10.10
Active ingredients		Number of appearances, N (%)	Mechanism of action		Average price per ounce
	Tocopheryl (vitamin E)	23 (58)	Downregulation of TYR gene and TYRP1/TYRP2		$12.52
	Niacinamide	8 (20)	Suppression of MITF; suppression of melanosome transfer		$13.81
	Salicylic acid	2 (5)	Epidermal remodeling; chemical peeling		$15.00
	Ascorbic acid (vitamin C)	16 (40)	Direct inhibition of tyrosinase		$15.78
	Kojic acid	3 (7.5)	Direct and indirect inhibition of tyrosinase, partly by downregulation of MITF and TYR		$24.89
	Titanium dioxide	1 (2.5)	Artificial pigment		$5.00
	No active whitening ingredients	9 (22.5)	N/A		$5.73

All 40 products had reviews mentioning product performance, 31 products had reviews mentioning ease of application, and 29 products had reviews discussing odor. Of 40 top products, 47.5% had reviews mentioning ingredients, seven of those products had exclusively positive reviews, seven exclusively negative, and five mixed. Products with exclusively positive comments regarding ingredients were rated slightly higher compared to negative or mixed (4.29 stars vs 4.22 stars). Twenty-nine out of 40 product reviews mentioned significant side effects, including rash, burning sensation, and discoloration. Only five products were recommended by a medical doctor. 

**Figure 1:**
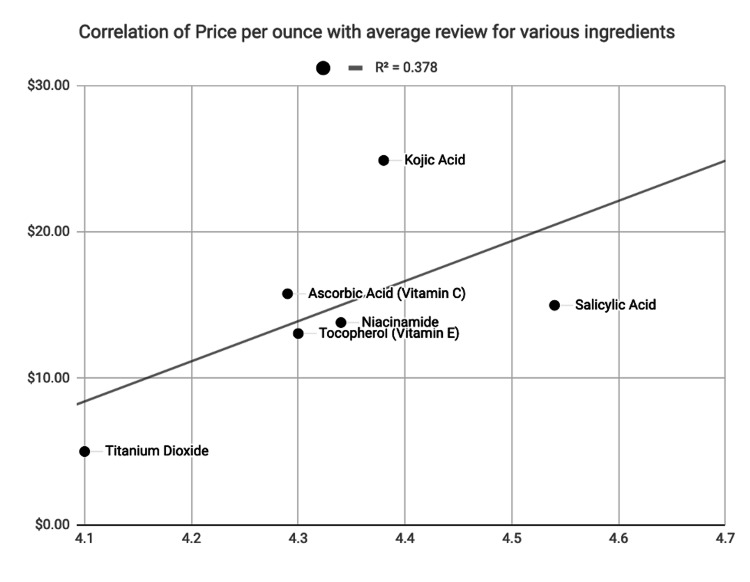
Correlation of price per unit of different ingredients. Association of cost per ounce and average review for products containing active skin-lightening ingredients. There is a moderate positive association between average consumer rating and cost, with R^2^=0.378.

Of the active ingredients listed in the product description, six ingredients were identified with a mechanism of action known to be associated with skin lightening either in vivo or in situ (Table 1). Of these, tocopheryl, or a form of it, was present in 58% of the top 40 products, ascorbic acid in 40%, and niacinamide in 20%. Notably, 22.5% of the top 40 products contained no known skin-lightening ingredient. There was a moderate positive correlation (R^2^=0.378) between price and consumer rating (Figure 1), with kojic acid being the most expensive per ounce ($24.89) and salicylic acid being the most highly rated (4.54 stars).

## Discussion

Facial hyperpigmentation is a highly prevalent concern, accounting for innumerable physician-patient interactions [5]. Skin pigmentation regulators include melanocytes in the epidermis that synthesize melanin, and keratinocytes that receive and distribute it in upper layers of the skin [6]. Other factors that may affect pigment cell function and proliferation and create alterations in pigmentation include fibroblasts in the dermis, endocrine factors, blood supply, neural factors, inflammation mediators, ultraviolet radiation, genetics, and skin injury [7]. Common types of hyperpigmentation are lentigines, melasma, and post-inflammatory hyperpigmentation. Wearing sunscreen and avoiding overexposure to the sun at an early age can help prevent the occurrence of many of these conditions. The skin appears as the most external and exposed anatomical region of the body, hence it is the first thing that people base their judgments on. An even skin tone and its luminosity is considered a major signal of a person’s attractiveness, health, and youth. Many studies have reported a link between skin texture and color with facial attractiveness [8-10]. Similarly, it has been found that an uneven facial skin color distribution, or tone can add up to 12 years of one’s perceived age [10]. 

Skin lightening has a long and complex history. While new technologies have brought lasers, complex acids, and procedures as safe and effective options for bleaching purposes, many products still contain adulterated and/or toxic products, such as mercury and lead. Easy accessibility to a wide variety of lightening agents available to the general public in OTC products can lead to overuse and increased risk for adverse effects since patients may assume that these products are safe to use without any supervision [11]. Despite warnings and bans due to adverse effects of many agents including even the gold standard of skin lightening, hydroquinone (HQ), the skin-lightening industry has experienced an exceptional growth and popularity all over the world in the recent years. The increasing interest in skin care over the last few years, and the rising preference for an even skin tone as a matter of confidence and enhanced beauty, has boosted the product demand. The global skin-lightening products market size was valued at 8.3 billion USD in 2018 [12]. By 2027, the skin-lightening industry is projected to be worth over $24 billion USD [4]. Furthermore, increasing the adoption of natural and organic products, which are considered to be safer, is expected to fuel the demand for these products over the forecast period.

Consumer’s inclination toward a single solution to different skin problems has increased the adoption of various skin lighteners into the market. HQ is considered the gold standard and is the most prescribed skin-lightening agent worldwide [13]. HQ is a phenol known to competitively inhibit melanin production by acting as a tyrosinase substrate. Through the release of semiquinone free radicals, melanocyte melanin production is damaged [14]. HQ is typically sold OTC in 2% concentrations, compared to 4% or higher concentrations in prescription products. Tocopheryl acetate, also known as vitamin E, is used for its powerful antioxidant, protective, and natural skin-conditioning properties. It is an important ingredient of many skin care products including lightening agents and has been in use for more than 50 years in dermatology [15]. However, vitamin E alone has shown minimal efficacy in the treatment of hyperpigmentation disorders such as melasma [16]. Vitamin C, kojic acid, topical corticosteroids, tretinoin, niacinamide, and salicylic acid are also among the most commonly used topical skin-lightening agents [14,17-19]. The actual efficacy of preparations containing only low percentages of these ingredients has not been fully elucidated [20]. It is important to note that many of the OTC skin-lightening products are cosmeceuticals containing pigments and other light-reflective compounds to create the appearance of lighter skin rather than having a permanent effect on skin color. Thus, as our study showed, many of these products lack a skin-lightening active ingredient. This explains findings from other studies showing that users of OTC skin-lightening products are frequently not satisfied with the results, reporting that OTC products did not improve their hyperpigmentation [11]. In addition, there is limited evidence-based research that demonstrates the effectiveness of natural ingredients as depigmenting agents [21].

Our study found that among the top 40 best-selling skin-lightening products on Amazon, approximately one fourth did not contain any active ingredient that interferes with melanogenesis. The most common products were vitamin E derivatives, followed by vitamin C derivatives. Interestingly, despite being recognized as the most effective skin-lightening agent, HQ only comprised 2% of products. It is likely that manufacturers avoid including ingredients like HQ - which would lead to the product being classified as an OTC drug rather than a cosmetic - to bypass FDA approval before releasing on the market [22].

The highest-rated active ingredient was salicylic acid ($15.00 per ounce). Kojic acid had the highest cost ($24.89 per ounce), which is much higher when compared to sunscreen (median price per ounce $3.32), the recommended first-line treatment of hyperpigmentation [23]. Titanium dioxide ($5.00 per ounce) was least expensive, though this ingredient does not have a true skin-lightening effect but rather is a pigment with sunscreen properties, which creates the appearance of lighter skin [24].

Finally, it is interesting that consumers prioritized “natural” ingredients. However, the term “natural” has not been defined by the FDA, making it more of a marketing term than a true indication of a product’s components [25].

## Conclusions

Our study is limited by a single retailer information and highly rated products, which makes the interpretation of the negative comments more difficult to generalize. Another important consideration is that the generalizability may be limited due to the lack of reviewer demographic information and possible lack of representation of the entire population. Although OTC lightening products are widely used, their overall effectiveness has yet to be established. Patients are often unsatisfied by their results when using these products. This could be due to the weaker composition of OTC products since higher percentages of certain ingredients like HQ need a prescription and/or FDA regulation along with physician supervision. Misuse of these agents is also another possible reason for poor outcomes in patients who use OTC products for lightening purposes. Tocopheryl, ascorbic acid, and niacinamide were the most popular ingredients. Notably, 22.5% of the top 40 products contained no known skin-lightening ingredient, another possible cause of low effectivity. Our study provides insight on the factors influencing the choice, preferences, and satisfaction of consumers who sought Amazon.com for the purchase of lightening products. Consumers appear to most value the product’s performance and ease of application when deciding upon product acquisition. It is important that dermatologists are aware of the availability and options of OTC products in order to properly educate and advise. A holistic approach that considers medical, cultural, and psychological factors should always be considered to develop adequate, well-tolerated, and beneficial treatment regimens. With the choice of multiple options also comes the ability to tailor clinical recommendations to each patient’s skin-lightening habits, health literacy, and lifestyle that can improve overall patient outcomes.
